# Longitudinal Weight Gain Trajectories After Initiation of Dolutegravir‐Based Antiretroviral Therapy: A Retrospective Cohort Study

**DOI:** 10.1155/arat/4740293

**Published:** 2026-06-26

**Authors:** Uraiwan Akanit, Tuanthon Boonlue, Prasittichai Poonphol, Theeranat Maneekanon, Phataranicha Donganon, Juthamas Suebsin, Nidtaya Dawvongyad

**Affiliations:** ^1^ Department of Pharmacy Practice, Faculty of Pharmaceutical Sciences, Ubon Ratchathani University, Warinchamrab, Ubon Ratchathani, Thailand, ubu.ac.th; ^2^ UBU Pharmacy Clinical Outcome Research (UPCOR) Group, Faculty of Pharmaceutical Sciences, Ubon Ratchathani University, Warinchamrab, Ubon Ratchathani, Thailand, ubu.ac.th; ^3^ Department of Pharmacy, Sunpasitthiprasong Hospital, Ubon Ratchathani, Thailand

**Keywords:** antiretroviral therapy, dolutegravir, integrase strand transfer inhibitors, people living with HIV, weight gain

## Abstract

Dolutegravir (DTG) is widely used in antiretroviral therapy (ART) for its high efficacy and favorable tolerability. However, emerging evidence has raised concerns regarding weight gain and potential metabolic consequences associated with DTG use, with limited longitudinal data from Asian populations. This study aimed to evaluate longitudinal weight changes among people living with HIV (PLWH) receiving DTG‐containing regimens and to determine whether weight trajectories differ across key demographic and clinical subgroups. We conducted a retrospective cohort study of adult PLWH who initiated DTG‐based ART at a tertiary referral center between January 2020 and June 2022, with follow‐up through June 2024. Participants with complete weight data at baseline, 6, 12, 18, and 24 months were included. Weight trajectories were analyzed using repeated‐measures ANCOVA, adjusting for sex, age group, ART backbone (TDF‐based vs. non–TDF‐based), baseline body mass index (BMI), baseline weight, and baseline CD4 cell count. A total of 157 participants (59.2% male; mean age 50.4 years) were included. Mean body weight increased significantly over 24 months in both sexes. Greater numerical weight gain was observed among participants aged ≥ 50 years and those receiving non–TDF‐based regimens. However, only ART backbone demonstrated a significant time‐by‐subgroup interaction. Participants with baseline CD4 counts < 200 cells/mm^3^ and lower baseline BMI demonstrated numerically greater weight increases over time. No significant time‐by‐sex, time‐by‐age, time‐by‐BMI, or time‐by‐CD4 interactions were observed, indicating broadly similar longitudinal trajectories across these subgroups after adjustment. DTG‐containing ART was associated with modest but significant weight gain over 24 months in this Thai cohort. ART backbone, differences between TDF‐based and non–TDF‐based regimens, was the primary factor associated with differential weight trajectories. These findings support monitoring of routine weight and metabolic parameters and highlight the importance of individualized ART selection to optimize long‐term metabolic health.

## 1. Introduction

Human immunodeficiency virus (HIV) continues to present a significant global health burden [1]. According to the UNAIDS 2022 report, the number of new people living with HIV (PLWH) has declined by 38% since 2010, from 2.1 million to 1.3 million in 2022. Similarly, AIDS‐related deaths have decreased by 69% since 2004 and 51% since 2010, falling to 630,000 in 2022. Among all PLWH, 86% are aware of their status, 89% are receiving antiretroviral therapy (ART), and 93% have achieved viral suppression, indicating substantial progress toward the UNAIDS 95–95–95 targets set for 2025 [2]. In Thailand, as of 2023, 580,000 individuals are living with HIV, with 470,000 receiving ART; however, 12,000 AIDS‐related deaths and 9100 new infections were recorded in the same year [3]. A nationwide study of over 398,000 Thai PLWH initiating ART between 2008 and 2021 showed a decline in AIDS‐related mortality from 60% to 50%, yet 43.6% began treatment with CD4 counts < 200 cells/mm^3^. Higher mortality was observed among women, those lost to follow‐up, and patients treated outside major cities, highlighting the need for earlier diagnosis and equitable access to HIV care [4].

The cornerstone of HIV treatment is lifelong ART, which suppresses viral replication, preserves immune function, and reduces HIV‐related morbidity and mortality. While effective, long‐term ART use is associated with potential adverse effects, necessitating ongoing pharmacovigilance and patient monitoring [5, 6]. A particular focus has emerged on managing metabolic side effects, as most first‐line regimens in Thailand now include dolutegravir (DTG), an integrase strand transfer inhibitor (INSTI), in combination with a nucleoside reverse transcriptase inhibitor (NRTI) backbone [7, 8].

Although DTG is highly efficacious and generally well tolerated, several studies have linked its use to weight gain, particularly among women and people of African descent [9]. While not all individuals experience significant weight changes, for some patients, weight gain may lead to further complications such as metabolic syndrome, Type 2 diabetes, and cardiovascular disease [10, 11].

In a Thai cohort study, virally suppressed PLWH who switched to INSTI‐containing regimens experienced significantly greater weight gain at 6, 12, and 18 months compared to those maintained on non‐INSTI regimens. Interestingly, weight trajectories among all regimens began to converge by 24 months [12]. However, despite these findings, there remains a paucity of data on specific risk factors associated with weight gain in Thai PLWH. Therefore, this study aimed to evaluate longitudinal weight changes before and after initiating DTG‐containing ART regimens in Thai PLWH patients. Additionally, we examined potential clinical factors associated with weight gain, including ART regimen type, baseline BMI, and CD4 cell count strata.

## 2. Materials and Methods

### 2.1. Study Design and Setting

This retrospective cohort study was conducted at a tertiary referral center in northeastern Thailand. The study included data from PLWH who initiated DTG‐based ART between January 1, 2020, and June 30, 2022, and were followed through June 30, 2024. Data collection covered baseline through 24 months of follow‐up.

### 2.2. Participants

Participants were retrospectively identified from the medical records. Inclusion criteria were as follows: (1) PLWH aged ≥ 18 years; (2) receipt of continuous care at the hospital for at least 24 months; and (3) availability of complete weight data at baseline. Exclusion criteria were as follows: (1) diagnosis of an opportunistic infection during the follow‐up period; (2) pregnancy at any point during treatment; or (3) missing baseline or follow‐up weights. Participants were identified using hospital electronic medical records, and follow‐up was conducted through routine clinical visits at 6, 12, 18, and 24 months.

### 2.3. Sample Size

A minimum sample size of 138 participants was calculated based on repeated measures ANCOVA (within‐subjects design) using G^∗^Power software, assuming a medium effect size (*f* = 0.25), *α* = 0.05, power = 0.90, and four postbaseline repeated measurements (6, 12, 18, and 24 months) in addition to baseline assessment. The selected effect size was based on Cohen’s conventional criteria for repeated‐measures analyses, in which *f* = 0.25 represents a medium effect size [13] considered appropriate for detecting clinically meaningful longitudinal weight changes in this setting. To account for potential exclusions due to missing data or loss to follow‐up, an additional 15% was added, resulting in the required sample size of approximately 158 participants. The sample size calculation was primarily intended to detect longitudinal within‐subject changes in body weight over time, which represented the primary study objective. Subgroup and interaction analyses were considered exploratory and may therefore have had reduced statistical power to detect smaller between‐group differences.

### 2.4. Data Sources and Variables

Data were abstracted using a standardized form from the hospital’s electronic medical records. Variables collected included the following: demographic characteristics (age and sex), clinical data (height, weight at five time points, body mass index [BMI], and baseline CD4 cell count), and ART regimen classification (TDF‐based regimens included TDF with 3 TC/FTC; non–TDF‐based regimens included ABC/3 TC/DTG, 3 TC/LPV/r/DTG, or other backbones without TDF). The primary outcome was change in body weight from baseline at 6, 12, 18, and 24 months. Exposures and subgroup variables included age group (18–49 vs. ≥ 50 years), sex, ART regimen type, baseline BMI category, and baseline CD4 category. All variables were obtained using uniform procedures from a single database to minimize measurement variation. To ensure longitudinal comparability across time points, only patients with complete weight data and continuous follow‐up were included in the primary analysis. Information bias was addressed by using standardized data abstraction procedures. Potential confounding was addressed through multivariable adjustment in repeated‐measures ANCOVA and linear mixed‐effects regression models.

### 2.5. Ethical Considerations

This study was approved by the ethics committee of the hospital (Reference No. 062/67R). The requirement for written informed consent was waived due to the use of anonymized retrospective data.

### 2.6. Statistical Analysis

Baseline categorical variables were compared using chi‐square or Fisher’s exact tests, as appropriate. Continuous variables were compared using Student′s *t*‐test or the Wilcoxon rank‐sum test according to variable distribution.

To evaluate longitudinal weight change over time, repeated‐measures ANCOVA was used. Covariates included sex, age group, ART backbone category, baseline BMI category, baseline body weight, and baseline CD4 category. Interaction terms between time and subgroup variables were additionally evaluated within linear mixed‐effects regression models with participant‐level random intercepts and robust standard errors to assess whether longitudinal weight trajectories differed according to sex, age group, ART regimen category, baseline BMI category, and baseline CD4 category. Weight change at each follow‐up time point (6, 12, 18, and 24 months) was calculated as the difference in weight from baseline (Δ weight = weight at time *t*–baseline weight).

All statistical tests were two‐tailed, and *p* values < 0.05 were considered statistically significant. Participants with missing baseline or follow‐up weight measurements were excluded from the primary complete‐case analysis. Sensitivity analyses using linear mixed‐effects models were additionally conducted to evaluate the robustness of longitudinal findings.

## 3. Results

**FIGURE 1:**
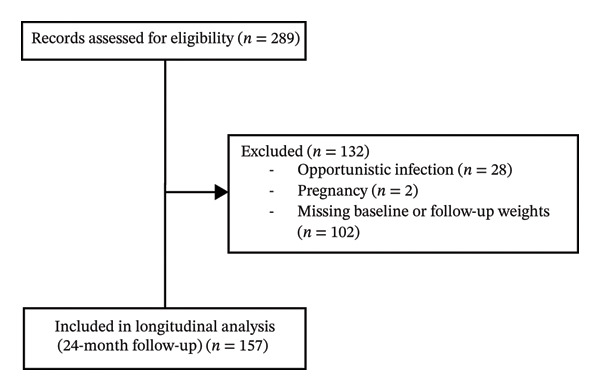
Flow diagram of the study.

A total of 289 PLWH initiated DTG‐containing ART between January 1, 2020, and June 30, 2022. After excluding 132 patients due to missing weight data (*n* = 74), early ART discontinuation or loss to follow‐up (*n* = 28), pregnancy (*n* = 2), or opportunistic infection (*n* = 28), 157 participants were included in the analysis as shown in Figure 1. A total of 157 participants were included in the baseline analysis, of whom 64 (40.8%) were female and 93 (59.2%) were male. The distribution of age groups did not differ significantly between sexes (*p* = 0.73). Similarly, there was no significant sex‐based difference in ART regimens, with comparable proportions of tenofovir‐based and non–tenofovir‐based regimens observed in females and males (*p* = 0.35). Baseline BMI categories showed a borderline difference between sexes (*p* = 0.069). Females had a higher proportion of underweight individuals (17.2% vs. 7.5%), whereas males more frequently fell into the overweight category (21.5% vs. 9.4%). The prevalence of obesity was comparable between females and males (29.7% vs. 23.7%). Baseline CD4 counts were similar between sexes, with no significant difference observed in the distribution of CD4 categories (*p* = 0.489) or in median CD4 levels (Wilcoxon rank‐sum test, *p* = 0.875). Additional demographic and clinical characteristics are detailed in Table 1.

**TABLE 1:** Baseline demographic and clinical characteristics of participants (*N* = 157).

Characteristics	Category	Male (*n* = 93)	Female (*n* = 64)	*p* value^∗∗^
Sex		93 (59.2%)	64 (40.8%)	—
Age group (years)	Younger adults (18–49 years)	41 (46.6)	30 (49.2)	0.73
	Older adults (> 50 years)	47 (53.4)	31 (50.8)	

ART regimen	Non–tenofovir‐based	62 (66.7)	38 (59.4)	0.35
	Tenofovir‐based	31 (33.3)	26 (40.6)	

Baseline body mass index (kg/m^2^)^∗^	Underweight (< 18.50)	7 (7.5)	11 (17.2)	0.069
	Normal weight (18.50–22.99)	44 (47.3)	28 (43.8)	
	Overweight (23.00–24.99)	20 (21.5)	6 (9.4)	
	Obese (≥ 25.00)	22 (23.7)	19 (29.7)	

Baseline CD4 count (cells/mm^3^)	Median [IQR]	435 [282–621]	505 [303–563]	0.875

Baseline CD4 group (cells/mm^3^)	< 200	15 (16.1)	9 (14.1)	0.489
	200–499	39 (41.9)	22 (34.4)	
	≥ 500	39 (41.9)	33 (51.6)	

^∗^BMI categories follow Asian‐specific cutoffs.

^∗∗^ *p* values were derived from the chi‐square test or Fisher’s exact test for categorical variables and Student′s *t*‐test or the Wilcoxon rank‐sum test for continuous variables, as appropriate.

### 3.1. Weight Changes Over Time by Gender, Age Group, ART Regimen, Baseline BMI, and CD4

In the overall cohort (*N* = 157), adjusted analyses demonstrated progressive weight gain over the 24‐month follow‐up period. Compared with baseline, the adjusted mean weight change was +0.15 kg (95% CI: −0.29 to 0.59) at 6 months, +0.43 kg (95% CI: −0.10 to 0.96) at 12 months, +0.91 kg (95% CI: 0.29 to 1.52) at 18 months, and +1.31 kg (95% CI: 0.68 to 1.93) at 24 months.

Over the 24‐month follow‐up period, mean body weight increased steadily in both sexes, with numerically greater gains observed in male participants. At 6 months, males had a mean weight change of +0.23 kg (95% CI: −0.10 to 0.55), and this increased to +1.33 kg (95% CI: 0.90 to 1.77) at 24 months. In females, weight increased from +0.04 kg (95% CI: −0.24 to 0.32) at 6 months to +1.26 kg (95% CI: 0.83 to 1.70) at 24 months. However, no significant time‐by‐sex interaction was observed (interaction *p* = 0.931).

When stratified by age group, participants aged ≥ 50 years demonstrated numerically greater weight gain throughout follow‐up, reaching +1.57 kg (95% CI: 0.81 to 2.33) at 24 months, whereas participants aged 18–49 years showed a weight change of +0.98 kg (95% CI: 0.02 to 1.95) at 24 months. Nevertheless, longitudinal weight trajectories did not significantly differ across age groups (interaction *p* = 0.828).

Participants receiving non–TDF‐based ART regimens demonstrated adjusted weight gains ranging from +0.39 kg (95% CI: 0.13 to 0.65) at 6 months to +1.05 kg (95% CI: 0.66 to 1.43) at 24 months. Participants receiving TDF‐based regimens demonstrated smaller changes during early follow‐up but greater increases at 24 months (+1.76 kg; 95% CI: 1.23 to 2.29). A significant time‐by‐ART regimen interaction was observed (interaction *p* = 0.024), indicating differential longitudinal weight trajectories according to ART backbone.

Differences in descriptive weight patterns were observed across baseline BMI categories. Underweight individuals (< 18.5 kg/m^2^) showed the highest increase at 24 months: +1.78 kg (95% CI: 0.71 to 2.86), while overweight participants gained the least weight and even experienced weight loss at 6 months (−0.98 kg; 95% CI: −1.49 to −0.46). Obese individuals showed minimal changes in 6 and 12 months, with a late gain of +0.96 kg (95% CI: 0.32 to 1.61) at 24 months. However, no significant time‐by‐BMI interaction was identified (interaction *p* = 0.379).

Participants with lower baseline CD4 counts demonstrated greater numerical weight increases over time. Participants with baseline CD4 counts < 200 cells/mm^3^ exhibited the largest adjusted gains, increasing from +0.92 kg (95% CI: 0.32 to 1.52) at 6 months to +2.79 kg (95% CI: 1.77 to 3.80) at 24 months. Participants with higher baseline CD4 counts demonstrated comparatively smaller weight changes over time. However, no significant time‐by‐CD4 interaction was observed (interaction *p* = 0.183). Full comparisons of weight change by gender, age group, and ART regimen are shown in Table 2.

**TABLE 2:** Adjusted estimated marginal mean changes in body weight over 24 months.

Subgroup	6 months mean‐difference (95% CI)		Mean ± SD	*p* value	12 months mean difference (95% CI)	Mean ± SD	*p* value	18 months mean difference (95% CI)	Mean ± SD		*p* value		24 months mean difference (95% CI)		Mean ± SD		*p* value	Interaction *p* value^∗^
Overall cohort (N = 157)	0.15 (−0.29 to 0.59)		60.29 ± 11.89	0.509	0.43 (−0.10 to 0.96)	60.57 ± 11.87	0.111	0.91 (0.29 to 1.52)	61.05 ± 12.07		0.004		1.31 (0.68 to 1.93)		61.45 ± 12.00		< 0.001	—
Gender																		0.931
Male	0.23 (−0.10 to 0.55)		63.18 ± 11.03	0.003	0.40 (0.03 to 0.77)	63.35 ± 11.17	0.075	0.99 (0.56 to 1.43)	63.95 ± 10.98	0.030		1.33 (0.90 to 1.77)		64.29 ± 11.27		0.054		
Female	0.04 (−0.24 to 0.32)		56.10 ± 11.80		0.48 (0.11 to 0.85)	56.54 ± 11.64		0.78 (0.36 to 1.20)	56.84 ± 12.29			1.26 (0.83 to 1.70)		57.32 ± 11.78				
Age group (years)																		0.828
18–49	0.04 (−0.73 to 0.80)		59.68 ± 11.07	0.664	0.06 (−0.80 to 0.93)	59.70 ± 10.83	0.219	0.53 (−0.41 to 1.48)	60.18 ± 10.96	0.273		0.98 (0.02 to 1.95)		60.63 ± 10.55		0.349		
≥ 50	0.24 (−0.26 to 0.74)		60.80 ± 12.57		0.74 (0.11 to 1.37)	61.29 ± 12.67		1.21 (0.46 to 1.97)	61.77 ± 12.93			1.57 (0.81 to 2.33)		62.12 ± 13.10				
ART regimen																		0.024
Non–TDF‐based	0.39 (0.13 to 0.65)		60.42 ± 12.14	0.002	0.52 (0.17 to 0.86)	60.55 ± 11.89	0.287	0.92 (0.54 to 1.29)	60.94 ± 12.00	0.817		1.05 (0.66 to 1.43)		61.07 ± 11.83		0.048		
TDF‐based	−0.28 (−0.69 to 0.14)		60.07 ± 11.38		0.28 (−0.14 to 0.69)	60.62 ± 11.78		0.89 (0.36 to 1.42)	61.24 ± 12.13			1.76 (1.23 to 2.29)		62.11 ± 12.21				
Baseline BMI (kg/m^2^)																		0.379
Underweight		0.75 (0.24 to 1.26)	45.55 ± 4.51	0.698	0.81 (−0.05 to 1.66)	45.61 ± 4.99	0.154	0.94 (0.01 to 1.87)	45.75 ± 5.33	0.085		1.78 (0.71 to 2.86)		46.59 ± 5.72		0.282		
Normal weight		0.46 (0.13 to 0.79)	55.39 ± 6.91		0.76 (0.42 to 1.10)	55.69 ± 6.95		1.47 (1.04 to 1.92)	56.41 ± 7.53			1.73 (1.28 to 2.19)		56.67 ± 7.22				
Overweight		−0.98 (−1.49 to −0.46)	64.31 ± 4.81		−0.51 (−1.08 to 0.04)	64.77 ± 5.85		−0.15 (−0.83 to 0.53)	65.13 ± 5.41			0.33 (−0.26 to 0.92)		65.62 ± 5.60				
Obese		0.05 (−0.40 to 0.51)	72.81 ± 11.29		0.29 (−0.33 to 0.92)	73.04 ± 10.61		0.56 (−0.06 to 1.19)	73.32 ± 11.10			0.96 (0.32 to 1.61)		73.72 ± 11.17				
Baseline CD4 group (cells/mm^3^)																		0.183
< 200	0.92 (0.32 to 1.52)		66.17 ± 13.38	0.025	0.83 (−0.22 to 1.69)	66.08 ± 12.09	0.559	2.33 (1.38 to 3.28)	67.58 ± 11.91	0.001		2.79 (1.77 to 3.80)		68.04 ± 11.93		0.001		
200–499	0.28 (−0.08 to 0.65)		59.09 ± 11.27		0.32 (−0.05 to 0.70)	59.13 ± 11.47		0.46 (0.05 to 0.87)	59.27 ± 11.39			0.91 (0.47 to 1.35)		59.72 ± 11.40				
≥ 500	−0.21 (−0.52 to 0.09)		59.35 ± 11.27		0.39 (−0.02 to 0.78)	59.96 ± 11.58		0.80 (0.34 to 1.27)	60.38 ± 11.94			1.14 (0.69 to 1.58)		60.71 ± 11.76				

*Note:* Mean differences represent adjusted estimated marginal mean changes (least‐squares means) derived from repeated‐measures ANCOVA models controlling for sex, age group, ART regimen, baseline BMI category, baseline body weight, and baseline CD4 category. Raw body weight values are presented as mean ± SD. ART, antiretroviral therapy.

Abbreviations: BMI, body mass index; CI, confidence interval; TDF, tenofovir disoproxil fumarate.

^∗^Interaction *p* values were derived from linear mixed‐effects models evaluating time‐by‐subgroup interactions with participant‐level random intercepts and robust standard errors.

### 3.2. Adjusted Longitudinal Weight Trajectories According to Demographic and Clinical Subgroups

**FIGURE 2:**
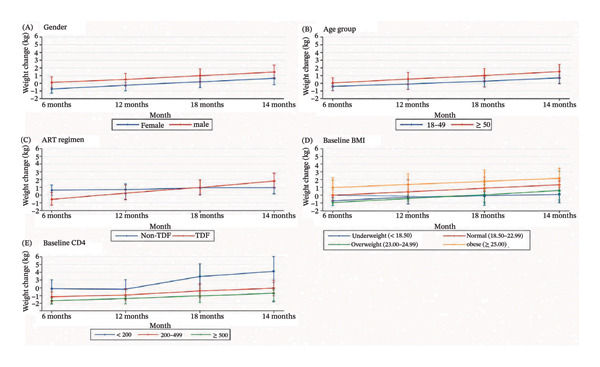
Adjusted longitudinal weight change over 24 months according to demographic and clinical subgroups. Values represent model‐based estimated marginal mean changes from baseline derived from mixed‐effects models with participant‐level random intercepts and robust standard errors. Models were adjusted for age, sex, baseline weight, ART regimen, baseline BMI category, and baseline CD4 category. (A) Gender. (B) Age group. (C) ART regimen. (D) Baseline BMI. (E) Baseline CD4.

Using linear mixed‐effects regression models with participant‐level random intercepts and adjustment for baseline weight, baseline BMI category, and baseline CD4 category, we observed a significant main effect of time on weight change across all subgroups. In gender‐stratified analyses (Figure 2A), adjusted trajectories for males and females rose in parallel with no significant time × sex interaction and no significant between‐sex differences over time. Age‐stratified models (Figure 2B) showed numerically greater adjusted weight gains among participants aged ≥ 50 years; however, no significant time‐by‐age interaction was identified (interaction *p* = 0.828). Regimen‐stratified analyses (Figure 2C) indicated differential longitudinal weight trajectories according to ART backbone, with a significant time‐by‐regimen interaction (interaction *p* = 0.024). Participants receiving non–TDF‐based regimens demonstrated greater adjusted increases during early follow‐up, whereas those receiving TDF‐based regimens demonstrated greater gains at 24 months. Baseline BMI strata (Figure 2D) showed the steepest adjusted increases among underweight and normal‐weight participants, while overweight participants demonstrated minimal or negative early changes. Nevertheless, no significant time‐by‐BMI interaction was observed (interaction *p* = 0.379). CD4 categories (Figure 2E) demonstrated greater adjusted gains among participants with baseline CD4 < 200 cells/mm^3^, particularly at 18 and 24 months. However, longitudinal weight trajectories did not significantly differ according to the baseline CD4 category (interaction *p* = 0.183).

## 4. Discussion

In this retrospective cohort study, individuals receiving DTG‐containing ART experienced a statistically significant increase in body weight over a 24‐month follow‐up period. Weight gain was observed across demographic and clinical subgroups; however, formal interaction analyses demonstrated differential longitudinal weight trajectories primarily according to antiretroviral backbone. Participants receiving non–TDF‐based regimens demonstrated greater adjusted weight increases during early follow‐up, whereas TDF‐based regimens showed greater gains at 24 months. Additionally, individuals aged ≥ 50 years demonstrated numerically greater weight gain during follow‐up; however, the adjusted time‐by‐age interaction was not statistically significant, suggesting broadly similar longitudinal weight trajectories between age groups after adjustment. Although male participants consistently had higher absolute body weight and numerically greater weight gain than females throughout follow‐up, formal interaction analyses demonstrated no significant time‐by‐sex interaction (*p* = 0.931), indicating that sex was not independently associated with differential longitudinal weight change after adjustment for baseline factors.

Our findings are consistent with prior research indicating that DTG use is associated with weight gain, particularly in the first 12 to 24 months of therapy. Randomized controlled trials such as ADVANCE [14] and NAMSAL [15] reported that ART‐naïve patients initiating DTG‐containing regimens gained more weight than those receiving efavirenz‐based therapy, with the greatest increases observed in regimens combining DTG with TAF and FTC. In the ADVANCE trial [14], for example, weight gain at 96 weeks reached 8 kg in the DTG + FTC/TAF arm, compared to 2 kg in the EFV + FTC/TDF arm. Similarly, Norwood et al. (2017) [16] found that patients switching to DTG gained 2.9 kg over 18 months, compared to 0.9 kg among those continuing efavirenz‐based therapy. However, in our cohort, the magnitude of weight gain was lower than that reported in these studies. A likely explanation is the absence of TAF in our regimens, as TAF was not covered by insurance during the study period. Prior comparisons of DTG combined with the two tenofovir prodrugs (TAF vs. TDF) [17] show that weight increases, encompassing both lean and fat mass, are greatest with TAF‐based regimens. Another contributing factor may be immune status as the mean baseline CD4 count in our sample was approximately 480 cells/mm^3^. Systematic reviews and meta‐analyses of weight change among PLWH receiving ART consistently show that lower baseline CD4 cell counts and higher baseline HIV RNA levels predict greater subsequent weight gain [18].

Although longitudinal weight trajectories did not significantly differ between sexes after adjustment, male participants consistently exhibited higher mean body weight and greater absolute weight gain than females over 24 months. Similar patterns have been described in some observational cohorts; however, several studies have reported that female sex is a stronger predictor of substantial weight gain following DTG initiation, particularly when combined with TAF [17, 19–21]. The apparent discrepancy across studies likely reflects heterogeneity in baseline anthropometry, ART backbone composition, and population characteristics. In Asian cohorts, lower baseline BMI and differences in body composition may reduce the magnitude of observable sex‐related differences in weight change, potentially explaining the broadly parallel weight trajectories observed in our adjusted analyses.

Older participants demonstrated numerically greater weight gain during follow‐up. Participants aged ≥ 50 years demonstrated consistently higher mean body weight and greater weight gain throughout follow‐up, with numerically greater adjusted weight gain observed throughout follow‐up. This finding aligns with a South African cohort study reporting greater weight increases among older individuals receiving DTG‐based therapy [22]. While some prior studies have identified younger age as a risk factor for ART‐associated weight gain [23, 24], our findings suggest that older individuals may demonstrate numerically greater weight increases in this setting, although longitudinal trajectories did not significantly differ after adjustment. Age‐related changes in body composition, reductions in physical activity, and higher baseline metabolic risk may contribute to these findings and warrant further investigation [25].

The comparison between TDF‐based and non–TDF‐based regimens is particularly notable. Although progressive weight gain occurred in both groups, participants receiving non‐TDF regimens demonstrated greater adjusted increases during early follow‐up, whereas TDF‐based regimens demonstrated greater weight gain at 24 months. These findings support accumulating evidence that TDF may exert a mitigating effect on ART‐associated weight gain when used with DTG [26]. Proposed mechanisms include mitochondrial toxicity and appetite suppression associated with TDF exposure [26, 27]. Conversely, discontinuation of TDF may unmask metabolic weight rebound. Observational studies have demonstrated increased weight gain following switches from TDF to TAF, including a French cohort reporting a mean gain of approximately 1 kg within 6 months of transition [28]. Similar patterns have been observed in East African populations, where weight gain accelerated after switching ART backbones, with more pronounced effects among women [29].

Differences in descriptive weight patterns according to baseline immunologic and anthropometric status were observed in this cohort. Participants with CD4 counts < 200 cells/mm^3^ had the greatest numerical weight increases over time, consistent with a “return‐to‐health” phenomenon following effective viral suppression, characterized by recovery from inflammation, improved appetite, and enhanced nutrient absorption [30]. In contrast, individuals with higher baseline BMI and body weight exhibited smaller gains, consistent with prior evidence suggesting that pretreatment nutritional status may influence ART‐related weight change. Integrase‐based regimens may amplify weight increases particularly among individuals initiating therapy with lower BMI and CD4 counts [30, 31].

Furthermore, lifestyle and hormonal factors may contribute to the variability in weight gain seen with DTG. A previous study [32] emphasized that hormonal contraceptive use, particularly depot medroxyprogesterone acetate (DMPA), may enhance appetite and promote weight gain through progesterone’s influence on metabolism. Other modifiable behaviors, such as high‐calorie diets and sedentary lifestyles, are also known to influence weight trajectories in PLWH [33], though such factors were not evaluated in this study.

This study is limited by its retrospective, single‐center design, which may introduce selection and information bias. In addition, the relatively modest sample size may have reduced statistical power for subgroup and interaction analyses. Key confounders, including diet, physical activity, concurrent medications, and metabolic parameters, were unavailable, and body composition was not assessed, precluding differentiation between fat and lean mass changes. Furthermore, metabolic outcome measures such as lipid profiles, glucose metabolism, insulin resistance, and cardiovascular risk markers were not available, limiting the ability to fully evaluate the clinical significance of the observed weight gain. Restriction to patients with complete follow‐up may further limit generalizability. Additionally, this study was conducted in a tertiary referral center that receives PLWH referred from multiple regional and community hospitals. As a result, 102 out of 289 initially screened participants (35.3%) were excluded because baseline or follow‐up weight measurements were unavailable within the institutional electronic medical record system. The inability to compare included and excluded participants may introduce selection bias and limit the generalizability of the findings. Therefore, the use of complete‐case analysis should be interpreted with caution.

Further research is warranted to clarify the mechanisms driving DTG‐associated weight gain, including natural longitudinal studies that incorporate dietary, hormonal, and genetic factors. Prospective trials comparing TDF‐, TAF‐, and ABC‐based backbones in combination with DTG should assess not only weight changes but also metabolic parameters, cardiovascular risk markers, and quality‐of‐life outcomes.

## 5. Conclusions

DTG‐containing ART was associated with a modest but significant increase in body weight over 24 months in this Thai cohort. Differences in weight gain trajectories were primarily observed according to antiretroviral backbone, with differential longitudinal weight trajectories identified between TDF‐based and non–TDF‐based regimens, supported by a significant time‐by‐regimen interaction. In contrast, no significant interactions were observed for sex, age, baseline BMI, or CD4 count, suggesting broadly similar longitudinal weight trajectories across these subgroups after adjustment. These findings support routine monitoring of body weight and metabolic parameters in patients receiving DTG‐based therapy and highlight the importance of individualized ART selection. Further prospective multicenter studies with comprehensive metabolic assessments are warranted to better characterize long‐term metabolic outcomes associated with DTG‐based therapy.

## Author Contributions

The authors confirm contribution to the paper as follows: study conception and design: Uraiwan Akanit, Prasittichai Poonphol, and Tuanthon Boonlue; data collection and investigation: Uraiwan Akanit, Phataranicha Donganon, and Theeranat Maneekanon; analysis and interpretation of results: Phataranicha Donganon, Theeranat Maneekanon, and Tuanthon Boonlue; project administration: Uraiwan Akanit and Tuanthon Boonlue; supervision: Nidtaya Dawvongyad and Juthamas Suebsin; visualization: Tuanthon Boonlue and Uraiwan Akanit; draft manuscript preparation: Uraiwan Akanit, Tuanthon Boonlue, Prasittichai Poonphol, Phataranicha Donganon, and Theeranat Maneekanon; critical review and editing: Uraiwan Akanit, Tuanthon Boonlue, Prasittichai Poonphol, Phataranicha Donganon, Theeranat Maneekanon, Nidtaya Dawvongyad, and Juthamas Suebsin.

## Funding

This research received no specific grant from any funding agency in the public, commercial, or not‐for‐profit sectors.

## Disclosure

All authors reviewed and approved the final version of the manuscript.

## Ethics Statement

This study was approved by the ethics committee of a tertiary care hospital, Ubon Ratchathani, Thailand (Reference No. 062/67R). As this was a retrospective study using anonymized data, the requirement for informed consent was waived by the ethics committee.

## Consent

Please see the Ethics Statement.

## Conflicts of Interest

The authors declare no conflicts of interest.

## Data Availability

The datasets used and/or analyzed during the current study are not publicly available due to institutional data protection policies but are available from the corresponding author upon reasonable request and with permission from the hospital.
